# Long-acting beta-agonists in the management of chronic obstructive pulmonary disease: current and future agents

**DOI:** 10.1186/1465-9921-11-149

**Published:** 2010-10-29

**Authors:** Donald P Tashkin, Leonardo M Fabbri

**Affiliations:** 1 David Geffen School of Medicine, Division of Pulmonary and Critical Care Medicine, UCLA, Los Angeles, California, USA; 2 Department of Respiratory Diseases, University of Modena & Reggio Emilia, Via del Pozzo 71, I-41124 Modena, Italy

## Abstract

Chronic obstructive pulmonary disease (COPD) is characterized by progressive airflow limitation and debilitating symptoms. For patients with moderate-to-severe COPD, long-acting bronchodilators are the mainstay of therapy; as symptoms progress, guidelines recommend combining bronchodilators from different classes to improve efficacy. Inhaled long-acting β_2_-agonists (LABAs) have been licensed for the treatment of COPD since the late 1990s and include formoterol and salmeterol. They improve lung function, symptoms of breathlessness and exercise limitation, health-related quality of life, and may reduce the rate of exacerbations, although not all patients achieve clinically meaningful improvements in symptoms or health related quality of life. In addition, LABAs have an acceptable safety profile, and are not associated with an increased risk of respiratory mortality, although adverse effects such as palpitations and tremor may limit the dose that can be tolerated.

Formoterol and salmeterol have 12-hour durations of action; however, sustained bronchodilation is desirable in COPD. A LABA with a 24-hour duration of action could provide improvements in efficacy, compared with twice-daily LABAs, and the once-daily dosing regimen could help improve compliance. It is also desirable that a new LABA should demonstrate fast onset of action, and a safety profile at least comparable to existing LABAs.

A number of novel LABAs with once-daily profiles are in development which may be judged against these criteria. Indacaterol, a LABA with a 24-hour duration of bronchodilation and fast onset of action, is the most advanced of these. Preliminary results from large clinical trials suggest indacaterol improves lung function compared with placebo and other long-acting bronchodilators. Other LABAs with a 24-hour duration of bronchodilation include carmoterol, vilanterol trifenatate and oldaterol, with early results indicating potential for once-daily dosing in humans.

The introduction of once-daily LABAs also provides the opportunity to develop combination inhalers of two or more classes of once-daily long-acting bronchodilators, which may be advantageous for COPD patients through simplification of treatment regimens as well as improvements in efficacy. Once-daily LABAs used both alone and in combination with long-acting muscarinic antagonists represent a promising advance in the treatment of COPD, and are likely to further improve outcomes for patients.

## Introduction

Chronic obstructive pulmonary disease (COPD) is a progressive disease characterized by increasing airflow limitation and respiratory symptoms, often associated with chronic comorbidities, leading to a significant burden for the patient.

Specific pharmacological therapy for COPD helps to prevent and control symptoms, reduce the frequency and severity of exacerbations, improve health status and improve exercise tolerance [1-3]. Inhaled bronchodilators, such as β_2_-agonists and muscarinic antagonists, improve lung function by altering airway smooth muscle tone but also act on peripheral airways [4]. They reduce air trapping and improve emptying of the lungs, thereby reducing lung volumes, improving symptoms such as breathlessness and increasing exercise capacity [1,5].

The present review focuses on the role and future of long-acting β_2_-agonists (LABAs) in the management of COPD, updating previously published reviews [6].

### β_2_-agonists

**Figure 1:**
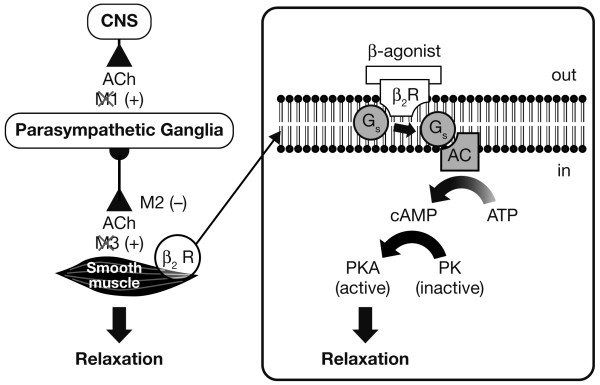
**Indirect and direct relaxation of smooth muscle**. Muscarinic antagonists (X) block M_3_ receptors to prevent binding of acetylcholine (ACh), indirectly stimulating smooth muscle relaxation via inhibition of bronchoconstriction. β_2_-agonists interact with β_2 _receptors (β_2_R) to activate coupling of the stimulatory G protein (G_s_) with adenylcyclase (AC). This leads to enhanced production of cyclic adenosine monophosphate (cAMP) which activates protein kinase A (PKA) and results in smooth muscle relaxation. β_2_-agonists may also interact with presynaptic β_2_Rs at parasympathetic ganglia, modulating parasympathetic neurotransmission.

The principal action of β_2_-agonists is to relax airway smooth muscle by stimulating β_2_-adrenergic receptors. This increases the intracellular messenger cyclic AMP that is responsible for the control of smooth muscle tone [7]. Thus, activation of the β_2_-adrenergic receptor results directly in bronchodilation. Muscarinic antagonists also facilitate bronchodilation but work by competing with acetylcholine for muscarinic receptors [8]. By inhibiting the action of acetylcholine at receptor sites in the lung, they indirectly inhibit contraction of airway smooth muscle. Figure 1[7,8] illustrates the pathways by which each class of bronchodilator produces smooth muscle relaxation. β_2_-adrenergic receptor agonists may also attenuate cholinergic neurotransmission due to stimulation of β_2_-adrenergic receptors on parasympathetic ganglia [9,10].

β_2_-agonists are widely used in the management of COPD, either alone or in combination with other bronchodilators, corticosteroids, or both. Short-acting β_2_-agonists (SABAs) were the first agents of the class to become available for the treatment of COPD. LABAs with a 12-hour duration of action were subsequently introduced in the late 1990s, following positive experience in asthma [11], providing improvements in bronchodilator efficacy and patient outcomes compared with SABAs which have a duration of action of only 4-6 hours [12]. The currently available SABAs and LABAs are summarized in Table 1[1,13-18].

**Table 1:** SABAs and LABAs commonly used in COPD

Drug	Dose delivered by inhaler (μg)	Other formulations	Recommended dose	Duration of action (hours)	Onset of action
Short-acting					
Salbutamol (albuterol)	100, 200 (MDI & DPI)	Solution for nebulizer; oral (syrup, tablets); vials for injection	200 μg up to 4 times daily	4-6	5 mins
Terbutaline	400, 500 (DPI)		500 μg up to 4 times daily	4-6	30 mins
Long-acting					
Formoterol	4.5-12 (MDI & DPI)	Solution for nebulizer (20 μg/2 mL)	12 μg twice daily (Aerolizer/Easyhaler 9 μg^† ^twice daily (Turbuhaler) 20 μg twice daily (Nebulizer)	12+	<5 mins*
Arformoterol		Solution for nebulizer (15 μg/2 mL)	15 μg twice daily	12+	6.7 mins*
Salmeterol	25-50 (MDI & DPI)		50 μg twice daily	12+	2 hours**

DPI, dry powder inhaler; MDI, metered dose inhaler * mean FEV_1 _increase of 15%; ** mean FEV_1 _increase of 12% or more and at least 200 mL; ^† ^delivered dose (equivalent to 12 μg metered dose)

### Current position in management guidelines

SABAs such as salbutamol (albuterol), terbutaline, pirbuterol or fenoterol and/or short-acting muscarinic antagonists (SAMAs) such as ipratropium bromide or oxitropium bromide are recommended as rescue medication for all severities of COPD to relieve acute symptoms of bronchospasm. For patients with Stage II (moderate) or more severe COPD, regular treatment with a twice-daily LABA such as formoterol or salmeterol, or a long-acting muscarinic antagonist (LAMA) such as tiotropium, is more effective and convenient than treatment with the shorter-acting agents [1]. As symptoms progress, combining bronchodilators from different classes is recommended to improve efficacy. Inhaled corticosteroids may be added to bronchodilator treatment in patients with severe COPD and a history of repeated exacerbations.

No one long-acting bronchodilator is recommended over another in management guidelines [1], because few studies have specifically compared different LABAs [19,20]. In addition, studies comparing LABAs with LAMAs have not been designed or powered to answer this clinical question to date, providing inconclusive results [21-23]. The choice therefore depends on availability and the response of the individual patient [1]. Inhaled therapy is preferred to oral therapy, in light of the greater risk of systemic side effects with oral beta-agonists and the unfavorable safety profile of oral methylxanthines.

Recently, there has been extensive research into the efficacy and safety of available twice-daily LABAs, as well as the development of new long-acting β_2_-agonists which have the potential for once-daily use [6]. This paper focuses on the current and future role of LABAs in COPD management. The efficacy and safety of currently available LABAs is reviewed, and their limitations considered. Articles were identified using a PubMed search including search terms for all currently available LABAs (salmeterol, formoterol, arformoterol), β_2_-agonists, efficacy, safety and chronic obstructive pulmonary disease/COPD. This is followed by the authors' views on the ideal criteria for a new LABA for COPD, and a discussion of the extent to which novel LABAs, currently being developed, may meet these criteria.

### Clinical efficacy

Efficacy in COPD has traditionally been assessed primarily through impact on lung function measured by spirometry. However, to adequately understand the impact of a medication, outcomes of importance to the patient including symptoms and quality of life must also be taken into account [24]. The following section reviews efficacy by endpoint to assess how far salmeterol and formoterol address the need to improve lung function, symptoms, exercise endurance, quality of life and exacerbations in patients with COPD. A summary is provided in Table 2[1,25-31].

**Table 2:** Summary of clinical efficacy for currently available β_2_-agonists in COPD

		Improvement in outcome				
	**Duration of action (hours)**^**1**^	Lung function	Breathlessness	Exercise endurance*	Quality of life	Exacerbations
Salbutamol (albuterol)	4-6	✓^1,25^	✓^1,25^	✓^1,25^	NA	NA
Salmeterol	≥12	✓✓^1,25-27^	✓^25-27^	✓^1,25^	✓(✓^‡^)^1,25-28^	✓✓^1,25,26,28^
Formoterol	≥12	✓✓^1,25,29^	✓✓^25,29^	✓^1,25^	✓✓^1,25,29^	✓^†1,25,29-31^

✓ evidence of effectiveness; ✓✓ evidence of effectiveness over SABA or SAMA; *Outcome demonstrated by all bronchodilators, lack of evidence of significant differences between them; ^† ^equivocal evidence depending on formulation; ^‡^evidence of numerical improvements over shorter acting comparator; NA = evidence not available

### Lung function

Formoterol and salmeterol have both demonstrated significant improvements in lung function [12,26,27,29-51].

Improvements in pre-bronchodilator forced expiratory volume in 1 second (FEV_1_) ranged from 50-90 mL compared with placebo [26,30,36,40,49]. Bronchodilation was rapid in onset with formoterol [34], although salmeterol had an onset of action slower than salbutamol or ipratropium bromide [44].

Improvements in lung function were sustained in studies of 3 months to 3 years' duration when twice-daily LABAs were used as maintenance therapy [28-31,46,48,51,52]. Some studies have suggested there may be a decline in bronchodilator efficacy over time with salmeterol [53,54]; over 6 months, significant declines in peak forced vital capacity (FVC) relative to placebo (-83 mL, p < 0.05), but not FEV_1 _(-12 mL), were observed with salmeterol [53]. Other studies have also suggested a partial loss of bronchodilator efficacy of formoterol over time. For example, the initial improvement in mean trough FEV_1 _(95% confidence interval [CI]) with formoterol 12 μg compared with placebo declined from 110 ml (90-130) after Day 1 to 70 mL (40-100) at Week 12 and to 50 ml (10-90) at Week 52 [55]. Moreover, there appears to be a partial decline in the integrated 12-hour improvement in FEV_1 _following the morning dose of formoterol 24 μg from 3 to 12 months of twice-daily therapy [46]. However, no evidence of clinically meaningful tolerance has been demonstrated in pooled or meta-analyses of salmeterol [56,57].

Further, in a post-hoc analysis from the 3-year Towards a Revolution in COPD Health (TORCH) study, salmeterol reduced the rate of decline in lung function by 13 mL/year compared with placebo (p = 0.003), which was not statistically different from the reduction in the rate of decline by salmeterol in combination with the inhaled corticosteroid fluticasone propionate [58].

Formoterol and salmeterol have demonstrated similar or greater improvements in lung function compared with ipratropium bromide [26,29,39,44,45] and significantly greater improvements compared with theophylline [35,46,59], but appear to be less effective than tiotropium [21,60].

### Symptom control

Numerous tools and questionnaires have been developed to assess respiratory symptoms in COPD ranging from patient rating of symptoms on diary cards, to validated scales such as the Transition Dyspnea Index (TDI) and Borg scale of perceived dyspnea, for which minimal clinically important differences (MCID) have been established [61-63].

Both formoterol and salmeterol have demonstrated significant improvement compared with placebo in diary card symptom scores including breathlessness, together with significant reductions in the requirement for rescue medication [12,23,27,29,30,32,36,37,40,48,50,51]. Improvements in symptoms were generally similar [26,27,46] or greater [29,59] with formoterol and salmeterol than with ipratropium bromide and theophylline.

Fewer studies have included the TDI instrument, and these have yielded inconsistent results [21,26,32,33,40,43]. Formoterol 18 μg twice daily, but not 4.5 μg or 9 μg twice daily via the Turbuhaler, significantly improved TDI by 1.15 units compared with placebo (1.77 vs 0.62 units; p = 0.002) [32], achieving the MCID (≥1 unit) for this instrument. Similarly, nebulized arformoterol (the active (R,R)-enantiomer of formoterol) 25 μg and 50 μg but not 15 μg twice daily significantly improved TDI (1.08 and 1.04 units vs placebo respectively) [33]. In contrast, salmeterol has not achieved the MCID for TDI in clinical studies [21,26,33,40,43]. Donohue et al [21] found that only 35% of patients receiving salmeterol achieved the MCID (p = ns vs placebo), compared with 42% receiving the LAMA tiotropium (p < 0.01 vs placebo).

### Exercise tolerance

There are fewer studies investigating the effect of LABAs on exercise tolerance. Two 12-week studies found no significant difference in distance covered in the incremental shuttle walking test with formoterol 18 μg twice daily via Turbuhaler compared with placebo [32,51]. Similarly, studies with salmeterol show inconsistent effects on walking tests compared with placebo [12,39,42,64]. However, in constant (submaximal) work rate exercise endurance tests (considered to be more responsive to the effect of pharmacotherapy) [63], significant improvements in exercise tolerance have been demonstrated for formoterol and salmeterol, accompanied by improvements in dynamic hyperinflation (exercise-induced air trapping which is a significant contributor to exercise limitation due to intolerable dyspnea) [65,66]. For example, in cycle tests performed at 75% of peak work rate, salmeterol significantly prolonged exercise time compared with placebo (6.0 ± 0.8 vs 4.5 ± 0.8 minutes, p < 0.05), although this fell just short of the 1.75 minute MCID proposed by Casaburi [67].

Data with active comparators are limited, but small studies have found similar improvements in exercise endurance and dyspnea ratings during submaximal or progressive exercise tests between twice-daily LABAs and ipratropium bromide [68-70].

### Health-related quality of life

In the majority of studies, significant improvements in health-related quality of life as measured by the St. George's Respiratory Questionnaire (SGRQ) have been demonstrated with formoterol compared with placebo [23,29-31,46] and with salmeterol compared with placebo [33,36,48,71] although other studies have failed to demonstrate a significant effect on quality of life [21,40,43,51].

In some [29,30,48,71], but not all [23,28,31,33,36] studies, improvements compared with placebo exceeded the MCID of 4 units for the SGRQ [72]. Although improvements in health-related quality of life with formoterol and salmeterol were at least as good as those with ipratropium bromide [26,27,29] and theophylline [59], overall, a meta-analysis found that LABAs produced a mean change from baseline of 3.36 units in SGRQ [73], falling short of the MCID for this instrument. Similarly, in a responder analysis of a 6-month study, only 42% of patients receiving salmeterol achieved the MCID for health-related quality of life, compared with 40% for placebo (p = ns) and 51% receiving tiotropium (p < 0.05 vs salmeterol) [21].

### Exacerbations

Exacerbations contribute greatly to the increased morbidity and diminished health-related quality of life of COPD patients [74]. However, there has been a lack of a consistent and widely accepted definition with which to evaluate and compare the efficacy of therapeutic interventions on this outcome [75]. Definitions of, and methods for counting, COPD exacerbations vary across studies, and statisticians disagree as to the most appropriate statistical method for analysis [76,77]. Thus, the results of clinical studies must be interpreted with caution.

To date, clinical studies have not been specifically designed to investigate the effect of formoterol on exacerbations, and no significant benefit on moderate to severe exacerbations has been demonstrated [23,30,31,78]. Salmeterol has more consistently demonstrated a significant effect on COPD exacerbation rate, which may reflect the study designs used [57]. The 3-year TORCH study found that salmeterol monotherapy reduced the annual rate of COPD exacerbations (requiring oral corticosteroids and/or antibiotics and/or hospitalization) by 15% compared with placebo (p < 0.001), although the greatest reductions were observed in the combination salmeterol/fluticasone propionate arm (25% annual reduction compared with placebo, p < 0.001) [28].

In a meta-analysis of 14 randomized clinical trials involving more than 6,400 patients, LABAs (salmeterol or formoterol) significantly reduced exacerbations requiring study withdrawal or hospitalization compared with placebo, with a relative risk of 0.78 (95% CI, 0.67-0.91) [73]. Hospitalizations tend to be relatively rare events and there may be large variability in the criteria applied by different physicians for hospitalizing a patient for an exacerbation, so this result is difficult to interpret. However, it is interesting to note that the use of ICS was not associated with a further risk reduction [73]. In a more recent meta-analysis of 23 randomized controlled trials of twice-daily LABAs (16 salmeterol vs placebo, four formoterol vs placebo, three salmeterol vs tiotropium and one formoterol vs tiotropium), LABAs significantly reduced the risk of an exacerbation compared with placebo (odds ratio [OR] 0.84; 95% CI, 0.76-0.92), whereas tiotropium significantly reduced the exacerbation risk compared with a LABA (OR 0.82; 95% CI, 0.72-0.93) [79]. A LABA-ICS combination tended to reduce the risk of an exacerbation more than a LABA alone, although this did not reach statistical significance (OR 0.90; 95% CI, 0.80-1.01).

### Efficacy summary

In summary, evidence to date suggests that both formoterol and salmeterol provide significant improvements in lung function, symptoms including dyspnea, and health-related quality of life. However, for the latter two endpoints, not all patients appear to achieve clinically meaningful improvements, and improvements were lower than those achieved with the once-daily LAMA tiotropium. Studies using the sensitive measure of submaximal exercise testing suggest there is a beneficial effect on exercise tolerance, although data are limited in this area. There are few definitive studies investigating the effect of treatment on exacerbations and results should be interpreted with caution, but suggest a significant effect, at least for salmeterol.

Given the general trend for twice-daily LABAs to provide improved lung function and more consistent symptom control compared with shorter-acting comparators (Table 2), one could argue that a longer duration of action is desirable in COPD [80]. The extension of this argument is that efficacy could be further improved by the introduction of a once-daily LABA.

### Clinical safety

There is clear consensus with regard to the central role of inhaled bronchodilators as the standard of care in COPD patients. However, recent concerns have been raised suggesting that LABA use could lead to an increased risk of respiratory death in patients with asthma. This was initiated by the results of the Salmeterol Multicenter Asthma Research Trial (SMART), which investigated the safety of adding salmeterol to usual asthma treatment in patients not already receiving a LABA; it was stopped following an interim analysis in 26,355 patients because of a small but significant increase in the number of asthma-related deaths in the salmeterol group [81]. These results raised significant doubts about the safety of LABA monotherapy in patients with asthma. Recent editorials have recommended that physicians should continue to use LABAs to treat asthma, but only when combined with appropriate doses of inhaled corticosteroids [82,83].

In COPD there are no restrictions on LABA use as monotherapy. COPD is a distinct disease from asthma, although there is some degree of overlap, with an estimated 10-50% of COPD patients having concomitant asthma. This wide variation in reported prevalence may relate to differences in diagnostic criteria [84,85]. An overlap in symptoms, together with the lack of validated discriminatory biomarkers, can make differential diagnosis difficult. The major distinguishing features between the two diseases are clinical: in general, COPD occurs later in life, with slowly progressive symptoms during exercise and is associated with a history of smoking or exposure to noxious gases and particulates. Asthma is likely to present early in life (often in childhood) and be associated with symptoms which vary from day to day, often at night or in the early morning; subjects often have a history of asthma in their family and airflow limitation tends to be more reversible than in COPD [1,86]. It is important to recognize that COPD, in most cases, is associated with significant, albeit never complete, reversibility [87-89] at one time or another. Therefore, it is not appropriate as is sometimes done, to refer to COPD patients who exhibit significant reversibility in response to a bronchodilator as having an 'asthmatic' component or 'mixed disease'. Indeed, the fact that most COPD patients respond significantly to a bronchodilator provides the major rationale for the recommendation of bronchodilators as the mainstay of therapy for COPD.

LABA monotherapy in COPD is not associated with increased risk of respiratory mortality. A meta-analysis performed in 2006 [90], suggested that β_2_-agonists were associated with an increased rate of respiratory deaths compared with placebo, and a 2-fold greater risk of severe exacerbations compared with muscarinic antagonists. However, this meta-analysis did not include the large dataset provided by the TORCH study [28]. TORCH found no increased risk of mortality with salmeterol monotherapy. Indeed, factorial analyses [91,92] have suggested the numerical mortality benefit observed with the combination (17.5% reduction in risk of death, p = 0.052) might be driven by the LABA component (Table 3) [91]. Further, a recent meta-analysis of 27 studies found no significant difference between LABA and placebo in terms of risk of respiratory death (relative risk 1.09 [95%, CI 0.45-2.64]) [73]. These results were confirmed in a more recent meta-analysis of 23 trials that also failed to find any increased mortality risk comparing a LABA with placebo (odds ratio 0.95 [95% CI, 0.71-1.27]) [79].

**Table 3:** Effects of treatment with salmeterol and fluticasone on mortality

					Main effect*			
Factor	Placebo (A)	Salmeterol (B)	Fluticasone (C)	Salmeterol plus fluticasone (D)	Salmeterol received		Fluticasone received	
					Yes (B+D)	No (A+C)	Yes (C+D)	No (A+B)
No. of subjects	1,524	1,521	1,534	1,533	3,054	3,058	3,067	3,045
No. of deaths	231	205	246	193	398	477	439	436
Probability of deaths at 3 years (%)	15.2	13.5	16.0	12.6	13.0	15.6	14.3	14.3
Hazard ratio (95% confidence interval)					0.81 (0.70-0.94)		1.00 (0.87-1.15)	
Chi-square					8.20		0.00	
P value					0.004		0.99	

*Data were analyzed according to a factorial design to estimate the main effect of each drug with adjustment for the other

Clinical studies have demonstrated that both formoterol and salmeterol are well tolerated, over periods of at least a year [28,30,31,36,48,78]. Commonly occurring LABA class effects include palpitations, headache and tremor, which may limit the dose that can be tolerated, especially in some older patients [1]. Cardiovascular side effects are also possible in susceptible patients [93], and there is some risk associated with the use of β_2_-agonists in patients with COPD and concomitant cardiac diseases, particularly chronic heart failure [94,95].

Chronic heart failure is a frequent comorbidity in patients with COPD. Cazzola et al [96] reported that COPD patients in Italy were at increased risk of chronic heart failure compared with the general population (7.9% compared with 2%), while in a Scottish study, 11.9% patients with COPD also had chronic heart failure [97]. Older individuals were at even higher risk in both studies. Moreover, heart failure may be under-diagnosed in COPD due to diagnostic confusion between the two diseases [98]. Research by Au et al. [99,100] indicates a strong trend towards a dose-response relationship of increased hospitalization and death, due to heart failure in patients with underlying heart failure who received increasing amounts of β_2_-agonist. Thus it is important that physicians should exercise caution in using high doses of β_2_-agonists in such patients.

However, studies specifically investigating safety have demonstrated that formoterol and salmeterol have good cardiovascular safety profiles [101,102]. Further, studies with formoterol found a similar profile irrespective of device or formulation [103,104]. In a large cohort of patients with no or stable cardiac comorbidities, the proportion of patients with treatment-emergent atrial tachycardia ranged from 27%-32% and was non-significantly higher, by 2%-5% (p = 0.70), in patients receiving salmeterol or arformoterol compared with placebo; more serious arrhythmias did not increase, nor did mean heart rate [105].

### Improving β_2_-agonists

Formoterol and salmeterol have acceptable benefit:risk profiles in the treatment of COPD; however, both have their limitations. For example, both formoterol and salmeterol have 12-hour durations of action and are approved for twice-daily dosing. The longer duration of bronchodilation of twice-daily LABAs compared with SABAs appears to be associated not only with improved lung function but also with more consistent efficacy in symptomatic endpoints, such as breathlessness and quality of life (Table 2). Evidence also indicates that LAMAs with a 24-hour duration of action have additional benefits in comparison with shorter acting SAMAs [106]. This suggests that sustained bronchodilation is desirable for a bronchodilator [80] and that a LABA with a 24-hour duration of action could provide further improvements in efficacy compared with twice-daily LABAs. In addition, patient adherence to treatment plans is a major obstacle to successful COPD management, especially since it is likely that patients will also be on a range of medications for comorbidities. Simplified dosing regimens are known to improve compliance [107], so that once-daily dosing is an important strategy to improve compliance and is a regimen preferred by most patients [6].

Fast onset of action is a significant feature of bronchodilators and may be beneficial for patients with COPD. Rapid relief of symptoms which patients can feel from the first dose provides reassurance of effect, and is likely to improve compliance with the medication [108,109]. When administered in the morning, the fast onset of action of bronchodilators is also advantageous, since COPD symptoms and patients' ability to perform daily activities appear to be worst in the morning [110]. While formoterol has an onset of action similar to salbutamol, salmeterol may not produce a significant increase in bronchodilation for as long as 2 hours following the dose.

For COPD patients, bronchodilator medication is a chronic maintenance therapy, and it is desirable that efficacy should be maintained over time. As discussed above there is some evidence of loss of bronchodilator effectiveness over time with β_2_-agonists, as demonstrated by Tsagaraki et al. [54], Donohue et al. [21], Dahl et al. [55] and Rossi et al. [46] although the clinical significance of these changes remains unclear.

Studies to date with twice-daily LABAs indicate that a minority of patients achieve clinically relevant improvements in dyspnea or health-related quality of life, based on current validated thresholds [21,73]. Data with current twice-daily LABAs in terms of preventing COPD exacerbations are mixed, with some studies showing no effect [30,31,48]. However, evidence from large datasets such as TORCH and pooled analyses reviewed above support a relatively modest reduction in the rate of moderate and severe exacerbations [28,73,79].

Improvements in these areas are desirable, but it is also essential that a new LABA has a comparable, if not better, safety profile compared with twice-daily LABAs. LABAs are associated with known class effects, the most significant being related to cardiac safety [1]. While the balance of evidence (as reviewed above) indicates that current LABAs are well tolerated, it is important that any new entry to the market ensures that potential cardiac effects are minimized, especially taking into account that COPD patients are often older and may have cardiovascular comorbidities.

Finally, consideration should be given to the delivery of any new molecule, ensuring the inhaler device is appropriate to the COPD patient. Many COPD patients have difficulty in using metered-dose inhalers so dry powder inhalers that are of low resistance, relatively insensitive to changes in airflow and can be used irrespective of inspiratory flow rate may be beneficial [111]. Soft-mist inhalers are also less technique-dependent than metered-dose inhalers [112], and may hold promise for future delivery of LABAs alone or in combination with other agents.

### Ultra-LABAs in development for use in COPD

'Ultra-LABA' is a term already in use to describe the class of once-daily LABAs in development [6,113]. Ultra-LABAs providing not only once-daily dosing but also improvements in the range of criteria outlined above would represent an advance on currently available LABAs, and an alternative to existing once-daily bronchodilators for the treatment of COPD. Tiotropium, the only once-daily inhaled bronchodilator currently available, is a LAMA that significantly improves lung function, increases exercise capacity and improves effectiveness of pulmonary rehabilitation, improves health status, and reduces exacerbations [1,79]. Although a four-year study of tiotropium on a background of usual therapy did not reduce the rate of decline in lung function [114], *post-hoc *analyses did demonstrate a modest, but significant, reduction in the rate of decline in lung function with tiotropium compared with placebo in patients not on other maintenance drugs [114,115]. However, tiotropium is relatively slow in onset of action and is associated with anticholinergic side effects, such as dry mouth (most commonly) and urinary retention (rarely) [1].

Ultimately, the choice of one agent over another as monotherapy in individual COPD patients will depend on a number of factors, including the expected side effects of each class of drugs, data on comparative efficacy with respect to onset, magnitude and duration of action, preference for the delivery device used and cost.

A number of novel LABAs with once-daily profiles are in development [6]. Here, we summarize the data available to date for the most promising of these.

**Figure 2:**
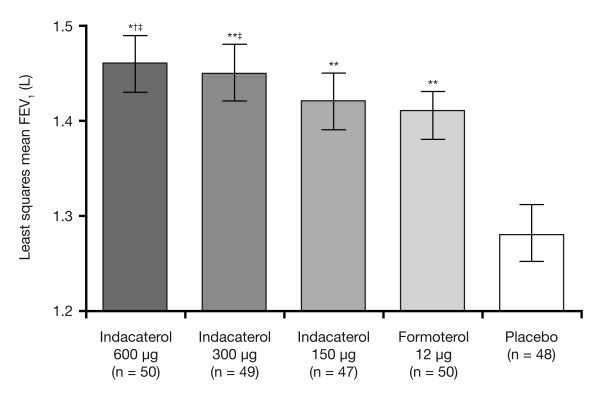
**Adjusted mean (±95% CI) standardized 24 hour trough FEV_1 _for indacaterol 150, 300 and 600 μg compared with placebo and formoterol 12 μg**. ANCOVA for treatment contrasts: **p < 0.001 vs. placebo; †p < 0.01 and ‡p < 0.05 vs. formoterol; ¶p < 0.05 vs. indacaterol 150 μg

*Indacaterol*, a novel inhaled ultra-LABA, has recently been approved in Europe for the treatment of COPD and is the first ultra-LABA to be launched in this region. Preclinical and Phase II studies indicated that indacaterol has a 24-hour duration of bronchodilation with fast onset of action, a good cardiovascular safety profile and no antagonism of the effect of rescue medication [22,116-121]. Further, Bauwens et al. [117] included doses approved for registration (150 and 300 μg) and demonstrated that the bronchodilator efficacy of indacaterol (150, 300 and 600 μg) at 24 hours post-dose was at least as good as formoterol 12 μg twice daily (Figure 2[117]).

It has been hypothesized that indacaterol's long duration of action may relate to its high affinity for the lipid raft domain of the cell membrane [122] (cholesterol-enriched microdomains of the lipid membrane, where β_2_-receptors are held together in close contact with signaling molecules and effectors) [123].

A number of placebo- and active-controlled Phase III studies in COPD have been completed or are ongoing [55,124-129]. Indacaterol 150 μg once daily significantly improved trough FEV_1 _at 12 weeks by 130-180 mL compared with placebo (p < 0.001) [125,127-129] while the 300 μg dose provided improvements of 170-180 mL (p < 0.001) [55,127,129], sustained over 1 year [55,129]. Both doses were significantly more effective than open-label tiotropium (p ≤ 0.01) [127] while in further studies, the 150 μg dose was significantly more effective than salmeterol 50 μg twice daily (p < 0.001) [128], and the 300 μg dose was significantly more effective than formoterol 12 μg twice daily (p < 0.05) [55].

Patient outcomes were also improved: indacaterol 150 and 300 μg significantly improved dyspnea as measured by TDI to a clinically relevant degree in a six month study (≥1 unit, p < 0.001) [127] with similar results achieved for indacaterol 300 μg throughout a 12 month study (1.00-1.32 unit improvement vs placebo, p < 0.001) [55]. In addition, indacaterol 150 and 300 μg were found to be at least as effective as open-label tiotropium in improving clinical outcomes over a 26-week period [127]. Indacaterol 150 and 300 μg also significantly reduced dyspnea compared with salmeterol and formoterol (p < 0.05) [55,128], and significantly reduced rescue medication use compared with tiotropium and salmeterol (p < 0.05) [127,128]. Patients reported significant improvements in health-related quality of life as measured by SGRQ for both doses compared with placebo in 6 month studies (p ≤ 0.01) [127,128] with the 150 μg dose achieving a clinically relevant improvement of 5 units versus placebo at 26 weeks in one study (p < 0.001) [128]. Further, indacaterol 300 μg achieved significant, clinically relevant improvements over one year (-4.7 units improvement at Week 52, p < 0.001 vs placebo) [55]. In studies of up to one year, indacaterol was well tolerated with an acceptable safety profile, including cardiovascular safety, similar to placebo, and comparable to tiotropium and formoterol [55,127,128].

*Olodaterol (BI-1744 CL) *is in a late stage of development. Preclinical studies with olodaterol suggested a potential for once-daily dosing in humans, along with a rapid onset of action and a favorable safety profile [130,131]. Phase III studies are based on doses of 5 and 10 μg once daily, delivered via the Respimat^® ^device [124].

*Carmoterol *has demonstrated long duration of activity in patients with COPD [132,133], with no tolerance observed over two weeks of treatment [134]. In a Phase II study, the 2 μg and 4 μg doses of carmoterol given once daily resulted in significant bronchodilation over 24 hours compared to placebo and comparable to that obtained with salmeterol 50 μg twice daily in patients with COPD [135]. No cardiac safety concerns were reported at doses less than 16 μg in healthy volunteers [136], while in patients with COPD initial data also suggests it has a good safety profile and is well tolerated [137].

*Vilanterol trifenatate (GW642444M) *[138,139] has also completed Phase II clinical trials [4,124]. In a four-week dose-ranging study involving 605 subjects, all doses (3, 6.25, 12.5, 25 and 50 μg once daily) achieved statistically significant increases in lung function (trough FEV_1_) compared to placebo (p < 0.05), with the two highest doses exceeding a predefined threshold of 130 mL increase in FEV_1_. Improvements in lung function were sustained over 24 hours. Vilanterol trifenatate was well tolerated at all doses and the frequency of adverse events was comparable to placebo [139].

*PF-00610355 *has been investigated in Phase I studies in healthy male subjects. A single dose of PF-00610355 450 μg had a superior duration of action as measured by specific airway conductance (sGAW) compared with placebo (16.4 hours longer) and salmeterol (9.8 hours longer) [140]. PF-00610355 was also well tolerated over 14 days within the anticipated clinical dose range (≤600 μg) [141]. Further studies will be needed to confirm if this drug has a 24-hour duration of bronchodilator action and is suitable for once-daily dosing.

### Potential impact of ultra-LABAs on COPD management as monotherapy and in combination

It could be hypothesized that a longer duration of bronchodilation with an ultra-LABA may be associated with superior and more consistent efficacy over a range of endpoints than is achieved with a twice-daily LABA. Furthermore, compared with complicated or multiple treatment regimens, simplified (once-daily) treatment regimens represent a significant convenience benefit and increase the likelihood of compliance with treatment - as does fast onset of action and a favorable safety profile. Together, these benefits could lead to improved overall clinical outcomes in COPD patients [6], and might help compliance with GOLD guidelines.

Based on the results of studies combining currently available LABAs and LAMAs [23,50,142-145], significantly greater improvements in lung function and symptoms can be achieved with a twice-daily LABA in combination with a LAMA compared with tiotropium used alone. Further, initial data from a single study suggests LABA/LAMA combinations may provide superior bronchodilator efficacy compared with LABA/ICS, at least in moderate COPD [146]; however, this was a short-term (6 weeks) study and did not assess possible differences in patient-reported outcomes. Longer-term studies are required with sufficient power to assess the relative efficacy of such combinations on quality of life and exacerbations. The introduction of ultra-LABAs is likely to further improve outcomes for patients, when used in combination with a LAMA. Current opinion is that it will be advantageous to develop inhalers containing combinations of two or more classes of once-daily long-acting bronchodilator drugs, in an attempt to simplify treatment regimens as much as possible [6]. Several ultra-LABA/LAMA combinations are in development, including QVA149 (indacaterol + NVA237 [glycopyrrolate]) [147,148] and vilanterol trifenatate/darotropium bromide [124].

As COPD progresses, patients with frequent exacerbations may require 'triple therapy' with a LAMA, LABA and an ICS. Evidence to date indicates that ICS/LABA in combination with tiotropium improves lung function, symptoms and health status, and reduces the risk of hospitalizations compared with tiotropium alone in patients with COPD [149-151]. These findings lend support to the concept that combining different classes of drugs with different mechanisms such as ultra-LABAs and once-daily LAMAs and ICS will further improve efficacy, and represent an important step towards the goal of optimal control for COPD patients.

## Conclusions

Long-acting bronchodilators are fundamental to the management of COPD; however, many patients remain symptomatic despite their use. Twice-daily LABAs salmeterol and formoterol provide some improvements in clinical outcomes compared with placebo and, although there are known class effects, have a favorable safety profile. However, in studies including validated instruments to measure dyspnea and health-related quality of life, not all patients achieve clinically meaningful improvements.

Thus, there is still an unmet need, particularly to improve symptoms, in many COPD patients. A range of features can be identified that could be considered to be criteria for a new LABA to fulfil. In particular, longer duration of action than 12 hours appears to be associated with improved, more consistent benefits across a range of outcomes. New ultra-LABAs have the potential to improve outcomes for COPD patients, both alone (providing an alternative to the only once-daily muscarinic antagonist bronchodilator currently available) or in combination. There are already promising data for indacaterol, which is likely to be the first ultra-LABA to be available for the treatment of COPD.

## List of abbreviations used

cAMP: Cyclic Adenosine Monophosphate; COPD: Chronic Obstructive Pulmonary Disease; DPI: Dry Powder Inhaler; FEV_1_: Forced Expiratory Volume in one second; FVC: Forced Vital Capacity; GOLD: Global initiative for Chronic Obstructive Lung Disease; ICS: Inhaled Corticosteroid; LABA: Long-acting β_2_-agonist; LAMA: Long-acting Muscarinic Antagonists; MCID: Minimal Clinically Important Difference; MDI: Metered-dose Inhaler; SABA: Short-acting β_2_-agonist; SAMA: Short-acting Muscarinic Antagonist; SGAW: Specific Airway Conductance; SMART: Salmeterol Multicentre Asthma Research Trial; SGRQ: St. George's Respiratory Questionnaire; TDI: Transition Dyspnea Index; TORCH: Towards a Revolution in COPD Health.

## Competing interests

DPT has received fees for serving on consulting/advisory boards from Boehringer Ingelheim, AstraZeneca, Dey Laboratories and Schering-Plough; speaker fees from Boehringer Ingelheim, Pfizer, Dey Laboratories and GlaxoSmithKline; and grant support from Almirall, Boehringer Ingelheim, Dey Laboratories, GlaxoSmithKline, Pfizer, Novartis and Sepracor.

LMF reports having served as a consultant to Nycomed, AstraZeneca, Boehringer Ingelheim, Chiesi Farmaceutici, GlaxoSmithKline, Merck Sharp & Dohme, Novartis, Roche and Pfizer; having been paid lecture fees by Abbott, AstraZeneca, Boehringer Ingelheim, Chiesi Farmaceutici, GlaxoSmithKline, Merck Sharp & Dohme, Novartis, Nycomed, Roche and Pfizer, having received grant support from Nycomed, Abbott, AstraZeneca, Boehringer Ingelheim, Menarini, Novartis, Schering Plough, Chiesi Farmaceutici, GlaxoSmithKline, Merck Sharp & Dohme, UCB, Pfizer, Italian Ministry of Health, and Italian Ministry for University and Research.

## Authors' contributions

Both authors contributed to the concept for the manuscript and critically reviewed and revised the initial and subsequent versions of the manuscript. Both authors read and approved the final version of the manuscript.

## Author details

DPT - David Geffen School of Medicine, Division of Pulmonary and Critical Care Medicine, UCLA, Los Angeles, California, USA; LMF Section of Respiratory Diseases, Department of Oncology, Haematology and Respiratory Diseases, University of Modena & Reggio Emilia, Policlinico di Modena, Via del Pozzo 71, I-41124 Modena, Italy.
